# Temperature and Thickness Dependence of the Thermal Conductivity in 2D Ferromagnet Fe_3_GeTe_2_

**DOI:** 10.1021/acsami.3c11578

**Published:** 2023-10-17

**Authors:** Marcel S. Claro, Javier Corral-Sertal, Adolfo Otero Fumega, Santiago Blanco-Canosa, Manuel Suárez-Rodríguez, Luis E. Hueso, Victor Pardo, Francisco Rivadulla

**Affiliations:** † CiQUS Centro Singular de Investigacion en Quimica Bioloxica e Materiais Moleculares, Departamento de Quimica-Fisica, Universidade de Santiago de Compostela, Santiago de Compostela E-15782, Spain; ‡ Department of Applied Physics, Aalto University, Aalto FI-00076, Finland; § Donostia International Physics Center (DIPC), San Sebastián E-20018, Spain; ∥ IKERBASQUE, Basque Foundation for Science, Bilbao E-48009, Spain; ⊥ CIC NanoGUNE BRTA, Donostia-San Sebastián E-20018, Spain; # Departamento de Física Aplicada, Universidade de Santiago de Compostela, Santiago de Compostela E-15782, Spain; ¶ Instituto de Materiais iMATUS, Universidade de Santiago de Compostela, Santiago de Compostela E-15782, Spain

**Keywords:** thermal conductivity, van der Waals, 2D materials, ferromagnetism, thermoreflectance

## Abstract

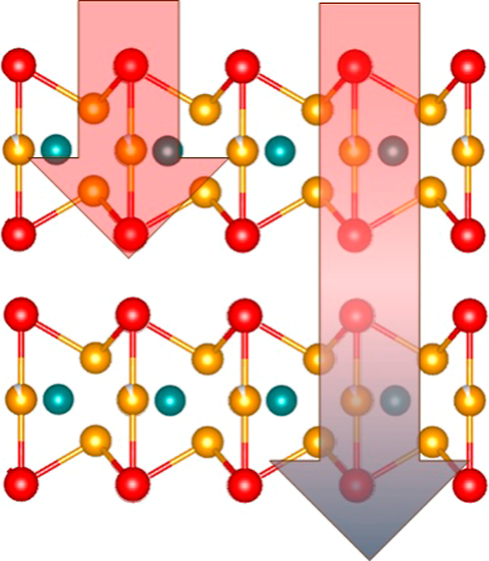

The emergence of symmetry-breaking orders such as ferromagnetism and the weak interlayer bonding in van der Waals materials offers a unique platform to engineer novel heterostructures and tune transport properties like thermal conductivity. Here, we report the experimental and theoretical study of the cross-plane thermal conductivity, κ_⊥_, of the van der Waals two-dimensional (2D) ferromagnet Fe_3_GeTe_2_. We observe an increase in κ_⊥_ with thickness, indicating a diffusive transport regime with ballistic contributions. These results are supported by the theoretical analyses of the accumulated thermal conductivity, which show an important contribution of phonons with mean free paths between 10 and 200 nm. Moreover, our experiments show a reduction of κ_⊥_ in the low-temperature ferromagnetic phase occurring at the magnetic transition. The calculations show that this reduction in κ_⊥_ is associated with a decrease in the group velocities of the acoustic phonons and an increase in the phonon–phonon scattering of the Raman modes that couple to the magnetic phase. These results demonstrate the potential of van der Waals ferromagnets for thermal transport engineering.

## Introduction

The electric-field control of the conductivity of atomic-thick graphene,^1,2^ shortly afterward extended to NbSe_2_ and MoS_2_,^3^ opened up new possibilities for material properties manipulation in the novel world of two-dimensional (2D) van der Waals (vdW) materials and heterostructures.^4^ Particularly, on vdW materials, the extreme bonding anisotropy is translated into a giant anisotropy also in the thermal transport, where the in-plane thermal conductivity κ_∥_ is much larger than the cross-plane one κ_⊥_,^5^ despite the prediction of phonon mean-free paths (mfp) of the order of several tens of nanometers across the weakly bonded planes.^6,7^ Defects and imperfect layer stacking result in a mixed contribution of ballistic transport (large mfp, coherent phonons) and diffusive transport (small mfp), which reduces very much the thermal conductivity across the 2D planes.^6,8^

Thermal transport is a crucial aspect for developing functional devices, which rely on an efficient heat dissipation to the base substrate, in a process determined by the thermal conductivity of the material itself and the thermal boundary conductance (TBC) of the interface with the substrate.^9,10^

A particularly interesting 2D material regarding heat dissipation is the itinerant ferromagnet Fe_3_GeTe_2_ (FGT): charge doping through Li^+^-intercalation modulates its magnetic anisotropy and increases *T*_C_ up to room temperature,^11^ while a strong spin-phonon coupling^12^ produces a significant effect of magnetic ordering on the thermal conductivity, opening the door to gate-tunable 2D thermal devices. First-principles calculations in other 2D magnetic materials, like 2H–VSe_2_, CrI_3_, FeX_3_, and RuX_3_ (X = Cl, Br, and I),^13−16^ have predicted a large change of the thermal conductivity in their magnetically ordered phase as well, although an experimental confirmation of such a large switching of the thermal conductivity associated with magnetic ordering in 2D materials is lacking.

In this work, we report experimental measurements combined with a theoretical analysis of the thickness and temperature dependence of the thermal conductivity in FGT. We have observed an increase in the cross-plane thermal conductivity with thickness, characteristic of a mixed ballistic propagation of long mfp phonons with diffusive transport, as well as a large drop in the thermal conductivity in the magnetically ordered phase. Both effects can be understood by our ab initio analysis of the thermal conductivity based on density functional theory (DFT) calculations.

## Results and Discussion

**Figure 1:**
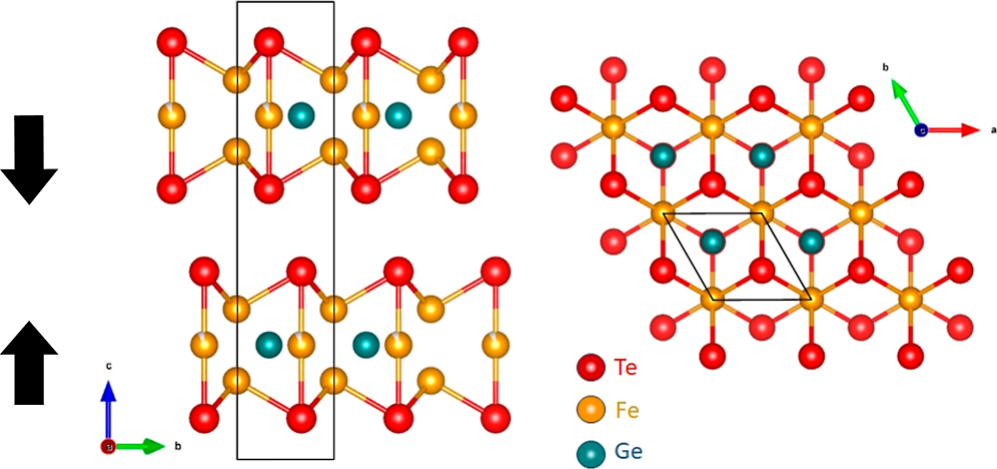
Crystal structure of Fe_3_GeTe_2_: left: lateral view of the structure. The cell is formed by two layers shifted with respect to each other. In the low-temperature magnetic phase, the layers become ferromagnetic (FM) with an antiferromagnetic interlayer coupling (as schematically shown by the arrows depicted). Right: top view of the structure showing the hexagonal symmetry of the *ab* plane. Fe, Ge, and Te atoms are shown in gold, purple, and green, respectively.

FGT is a 2D itinerant ferromagnet with *T*_C_ ≈ 200 K, which decreases with the number of layers but retains the magnetic order down to the single-layer limit.^17^ Neutron diffraction data support a ferromagnetic (FM) order also along the *c*-axis,^18^ although theoretical calculations and analysis of experimental magnetic susceptibility suggested an antiferromagnetic (AF) stacking below *T*_C_ ≈ 152 K.^19^ From the structural point of view, the material is weakly bonded off the plane via van der Waals interactions, which facilitates its mechanical exfoliation and transfer of few layer thick flakes to a substrate. The unit cell consists of two vdW planes with hexagonal symmetry, each formed by 3 Fe atomic planes (see Figure 1).

**Figure 2:**
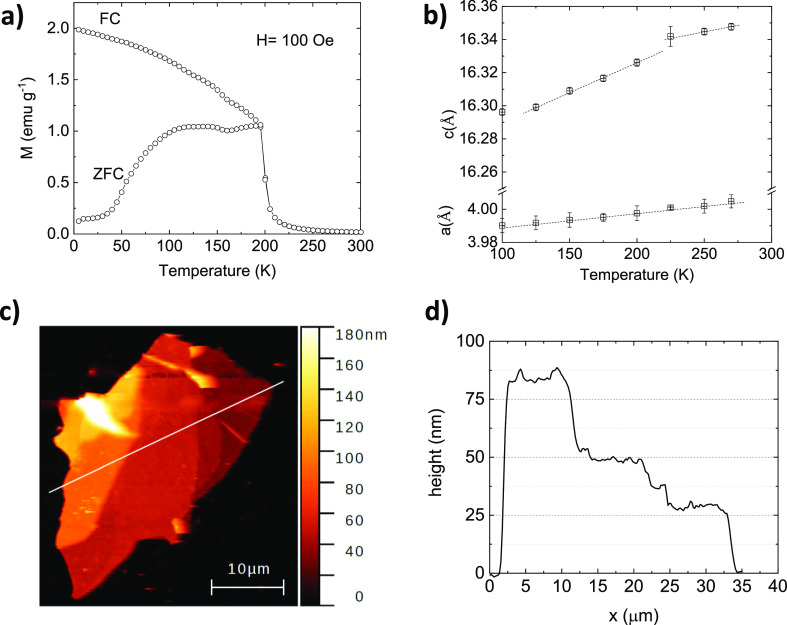
(a) Temperature dependence of the magnetization zero-field (ZFC) and field cooling (FC) curves measured at *H* = 100 Oe, and (b) lattice parameters measured for a bulk crystal of FGT. (c) Atomic force microscopy (AFM) image of one flake transferred to the surface of a (0001) sapphire substrate. The corresponding height profile along the line in panel c is shown in panel (d).

The crystals for this study were exfoliated from larger pieces obtained from HQ graphene (see Supporting Information for further details of the structural and chemical characterization of the samples). DC magnetization data of bulk crystals show that *T*_C_ ≈ 200 K, as expected for fully stoichiometric crystals (Figure 2a shows how the zero-field-cooled and field-cooled magnetization curves separate from each other at the transition temperature). Temperature-dependent X-ray analysis shows a change in the slope of the *c*-axis parameter at *T*_C_ (Figure 2b), but no change in the space group of the crystal accompanies the transition.

Few layer thick flakes of FGT were prepared by mechanical exfoliation and transferred to (0001) sapphire substrates using PDMS stamping.^20^ Transferred FGT flakes have tens of microns in lateral dimensions and thicknesses ranging from 15 to 250 nm (Figure 2c,d; see also Supporting Information). The flakes are always thicker than 5 layers, considered to be the border between 2D and 3D magnetism,^21^ so that the comparison with the bulk calculations is justified.

Thermal conductivity was measured by Frequency Domain Thermoreflectance (FDTR), using a ≈60 nm thick layer of Au as a transducer^22^ (see Figure S4 in the Supporting Information). To extract κ and the TBC from the FDTR phase-shift curves, we fitted the most common model wherein total energy conservation and energy transfer between layers are imposed by a transfer matrix, as explained elsewhere^23^ and in the Supporting Information. The multiparameter fitting can lead to unrealistic results if several parameters are kept free and the initial guess is distant from the global minimum. To reduce the number of fitting parameters, the thickness of the Au layer was measured by X-ray reflectivity, and its thermal conductivity was estimated from the sheet electrical resistance measured by the van der Pauw method and the Wiedemann–Franz law. The thickness of the FGT flakes was measured by atomic force microscopy (AFM). Heat capacities were taken from the literature, confirmed by differential scanning calorimetry (DSC), and kept fixed for each temperature in all fittings.^24^ The thermal conductivity of the substrate was measured and confirmed with the values from the literature.^25^ Since vdW materials present high anisotropy between the conductivity in-plane κ_∥_ (κ_*xx*_, κ_*yy*_) and cross-plane κ_⊥_ (κ_*zz*_), their values could be considered separately in the model. However, the sensitivity to κ_∥_ is very low, and it has a negligible influence in the κ_⊥_ value (see Supporting Information Figure S4 for the sensitivity analysis). Thus, κ_∥_ = κ_⊥_ was assumed. In this way, the free parameters in the fittings are reduced to κ_⊥_ of FGT and the TBC between Au/FGT, G1, and between FGT/Al_2_O_3_, G2. We considered the initial values for G1 ≈ 30–40 MW m^–2^ K^–1^, similar to Au/MoS_2_,^6^ and G2 ≈ 25 MW m^–2^ K^–1^, as reported for MoS_2_/Al_2_O_3_,^26^ and MoS_2_/SiO_2_^6^ interfaces.

On the other hand, the variability of G2 between mechanically transferred flakes may be an important source of error. For that reason, multilayer flakes like those shown in Figure 2c are important to reduce problems associated with the variability of G2, as they allow the measurement of κ_⊥_ for different thicknesses with the same FGT/sapphire interface (see also Figure S3 in the Supporting Information).

**Figure 3:**
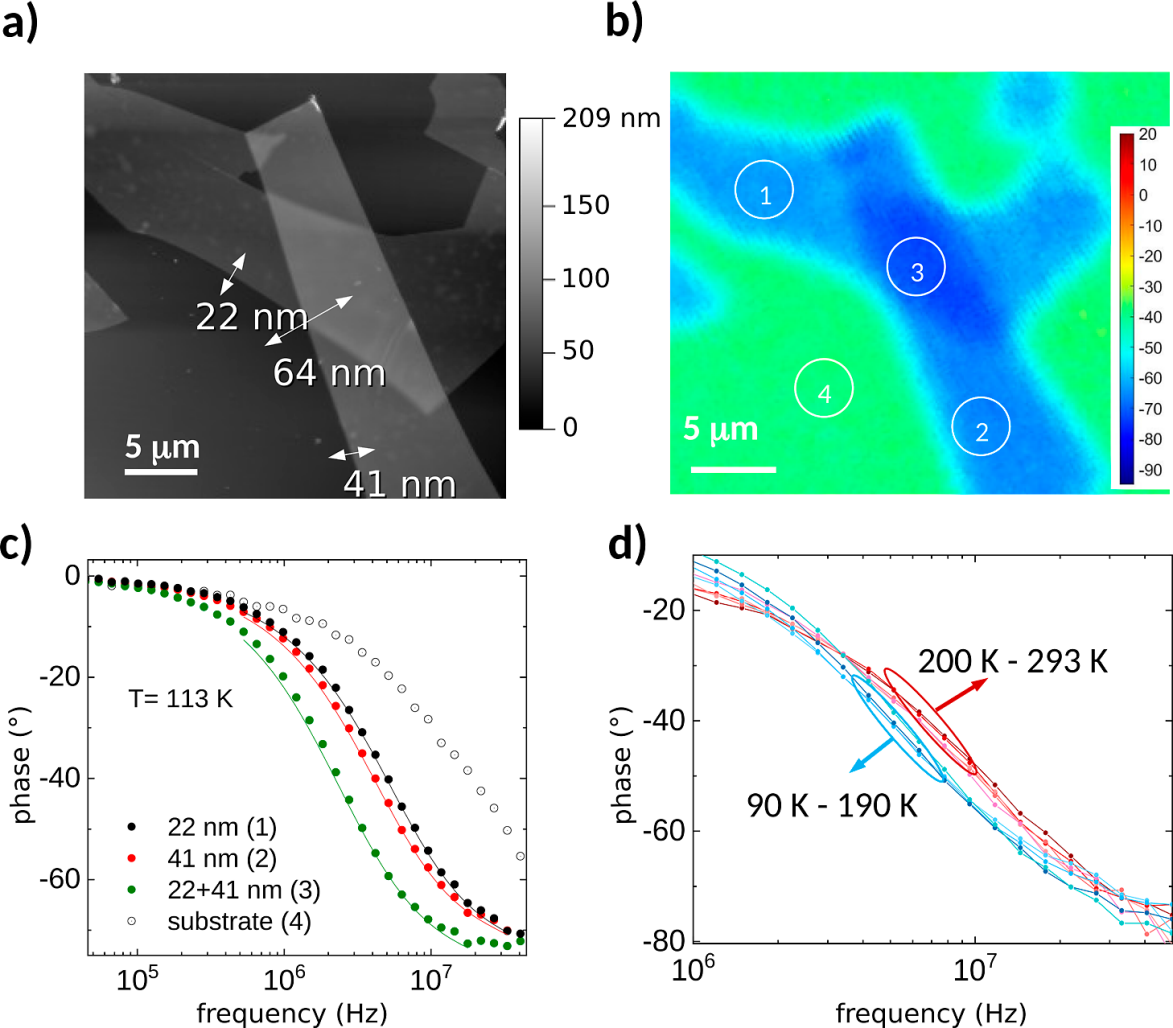
(a) 30 × 30 μm AFM topography of two partially overlapping flakes. (b) Phase-shift map at 20 MHz of the same region (enclosed within the square) observed in (a), with the corresponding points marked. In this image, the flakes are already covered with 60 nm of Au for the FDTR measurements. (c) Phase-shift vs frequency curves for the three points marked in (a,b), along with the fitting to the thermal model. The curve of the substrate, as a reference, is also shown. (d) Phase-shift vs frequency curves of point 2 at different temperatures. There is a large change around 200 K associated with the magnetic ordering temperature (see text).

Initial values of κ_⊥_ of 1 Wm^–1^ K^–1^, typical for other 2D materials, were used for an estimation of the sensitivity to different parameters in different frequency ranges. The spot size was varied between a 1/*e*^2^ diameter ≈ 4 and 11 μm for achieving better sensitivity to TBC and κ_⊥_. The fittings shown in Figure 3c to obtain the κ_⊥_ and TBCs reported in this work were performed from 1 to 50 MHz, where the sensitivity for these parameters is maximum (see Figure S4 in the Supporting Information).

Figure 3a shows the AFM topography of two partially overlapping flakes of thicknesses 22 and 41 nm, respectively. The 30 × 30 μm phase shift map at 20 MHz shows the variations in the contrast due to the differences in κ_⊥_ and TBC. The whole frequency phase-shift spectra for each point marked in (a,b) are presented in Figure 3c,d at different temperatures, demonstrating good sensitivity to thickness and temperature.

**Figure 4:**
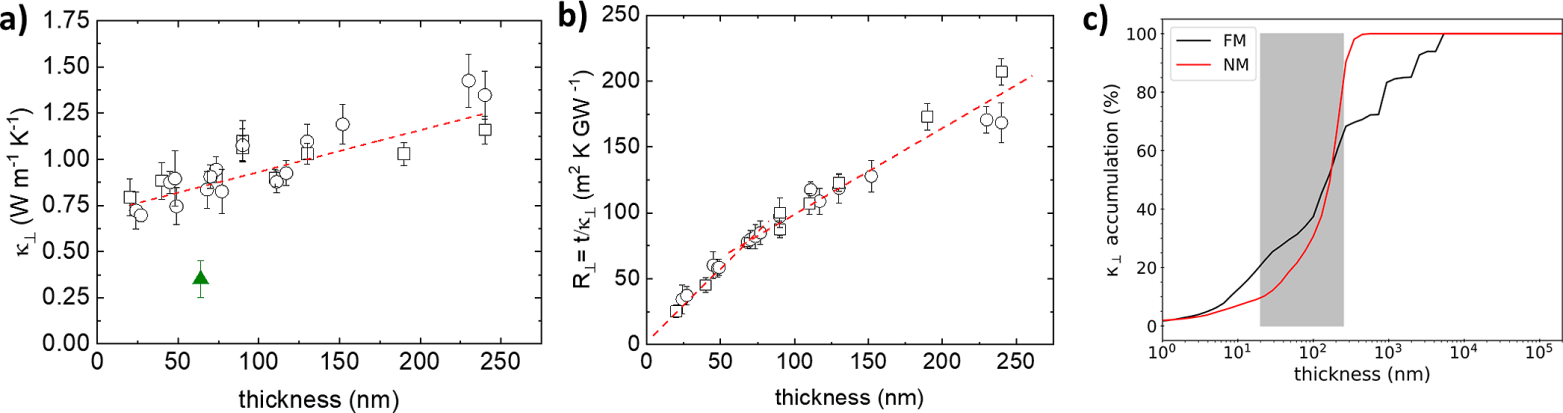
Measured thermal conductivity (a), and thermal resistance *R*_⊥_ = *t*/κ_⊥_, (b) at room temperature for different flakes with varying thicknesses. Circles and squares correspond to different sets of crystals transferred to different substrates. The green solid triangle in (a) corresponds to κ_⊥_ at point 3 in Figure 3a, the region of superposition of the two flakes. The dotted lines are linear fittings. (c) Accumulated κ_⊥_ as a function of the phonon mean free path at 300 K. The shaded area shows the thickness range of the flakes studied by FDTR in this work.

The thickness dependence of κ_⊥_ at room temperature is shown in Figure 4a. To reduce errors from sample preparation and defects, several flakes were measured, and each flake was measured several times. Thus, the error bars represented in the figure are obtained from the statistical variance. In this figure is observed an increase of κ_⊥_ with the thickness of the sample, of the order of ≈0.5 W/m K in a range of ≈200 nm. Although small, this is of the same order of magnitude as that reported for other van der Waals materials, like MoS_2_^6^ or SnSe_2_,^27^ and it is consistent with our DFT calculations for FM and nonmagnetic (NM) phases (Figure S11 of Supporting Information). The calculated accumulated κ_⊥_; Figure 4c) shows that more than 50% of the heat at 300 K is carried by phonons with a mean-free path larger than ≈200 nm, suggesting an important contribution from ballistic phonons along the *c*-axis, as in other vdW structures.^6,28^

In the case of pure ballistic transport, phonons can propagate without thermal resistance inside the material so that *R*_⊥_ = *R*_int_ + *t*/κ_⊥_ should be a constant, independent of thickness. However, the measured experimental cross-plane thermal resistance, *R*_⊥_, also increases with the thickness (Figure 4b). On the other hand, in a purely diffusive regime, *R*_⊥_(*t*) is linear with a constant slope = 1/κ_⊥_.^8^ For FGT, *R*_⊥_ increases linearly with thickness above ≈60 nm, giving κ_⊥_ ≈ 1.9(1) Wm^–1^ K and *R*_int_ ≈ 46 m^2^ K/GW, but it deviates from this behavior for thinner samples, with a vanishing resistance as *t* → 0. The change in slope suggests some thickness-dependent contribution, and although the data in Figure 4b seem to extrapolate to zero, the thinner samples measured in this work are *t* ≈ 25 nm, so we cannot exclude a small finite value of *R*_⊥_ close to the monolayer limit (note that a residual value as small as ≈10 m^2^ K/GW has been reported for a few monolayers of MoS_2_).^6^

We have also measured κ_⊥_ in the superposition region of two crystals, point 3 in Figure 3a: κ_⊥_ is substantially reduced in the overlapping region of total thickness 63 nm (green triangle in Figure 4a). Actually, the phase-shift curve of point 3 can be fitted with two layers, of 22 and 41 nm each, with their corresponding κ_⊥_, and a high interlayer thermal resistance between both flakes of ≈180 m^2^ K/GW (TBC ≈ 10–12 MW m^–2^ K^–1^). The value of the TBC between the two FGT flakes is of the same order of magnitude as reported for interfaces between dissimilar 2D materials, like graphene/MoS_2_ or MoS_2_/WSe_2_,^9^ although in this case, the large interfacial resistance occurs between films of the same composition without any mass density or compositional mismatch.

**Figure 5:**
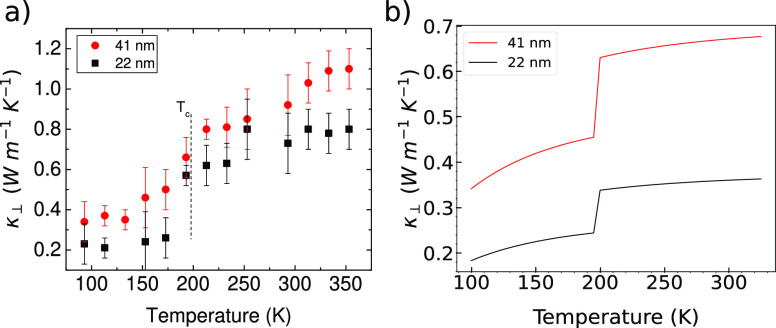
Experimental (a) and theoretical (b) temperature dependence of the thermal conductivity of two flakes with thicknesses 22 and 41 nm, corresponding to points 1 and 2 in Figure 3a, respectively. (b) Calculated temperature dependence of κ_⊥_ for the NM and FM phase of bulk FGT, considering the NM (FM) phase above (below) the experimental value of the magnetic transition temperature.

Finally, the experimental temperature dependence of κ_⊥_ is shown in Figure 5a for two different thicknesses (points 1 and 2 in Figure 3a). A reduction of κ_⊥_ between 25 and 65% occurs below *T*_C_ in the transition to the magnetic phase. Note that the jump in κ_⊥_ is clearly observed in the raw phase-shift curves (Figure 3d) and, therefore, cannot be attributed to fitting artifacts.

It is common in magnetic and ferroelectric materials that the formation of domain walls causes a reduction of thermal conductivity due to phonon scattering on domain boundaries.^29,30^ However, the κ_⊥_ obtained is robust to external magnetic fields up to 50 mT, applied with a strong toroidal permanent magnet (see Supporting Information Figures S7 and S8). Based on previous reports,^31^ this applied field should be enough to switch between stripe domains and uniformly magnetized states; the negligible effect of the magnetic field on κ_⊥_ indicates that the in-plane magnetic domains are not the cause of the sudden change of κ_⊥_ at *T*_C_. We have also discarded as a cause of the jump on κ_⊥_ the eventual changes in the crystal structure since the powder X-ray diffraction of the original bulk crystal revealed only a small change in the *c*-axis lattice parameter and thermal expansion without any crystallographic transformation (Figure 2b). Below *T*_C_, magnons could be an additional source of heat flow, providing an increase in the thermal conductivity; however, the opposite trend is found experimentally, suggesting that heat transport by phonons is the dominant effect in this system, at least for κ_⊥_.

In order to shed light on the observed experimental behavior, we carried out DFT-based calculations on the system. We have studied several magnetic orderings in the system and found that the ground state is the solution where FM layers couple antiferromagnetically. We have computed the temperature dependence of κ_⊥_ in the small-grain limit^32^ for the different experimental cases (Figure 5b). The thickness of the flakes was used as the boundary length for the 41 and 22 nm flakes. To identify possible changes at the transition, we have modeled differently the system for temperatures above and below *T*_C_. The ground-state FM solution was considered below *T*_C_, but above *T*_C_, we will consider a nonmagnetic (NM) solution as a possible proxy for the disordered paramagnetic phase above the Curie temperature. Our calculations show an ≈30% drop in the thermal conductivity between the NM and FM phases in good agreement with the experimental observations. Note that the theoretical underestimation of the thermal conductivity is related to the limitations of the small-grain limit used in the calculations in which the boundary scattering is overestimated, especially at higher temperatures and for larger samples. However, all of the qualitative features are well captured (the change at the transition and also the thickness dependence). Further details about the thickness dependence of the calculations can be found in the Supporting Information.

**Figure 6:**
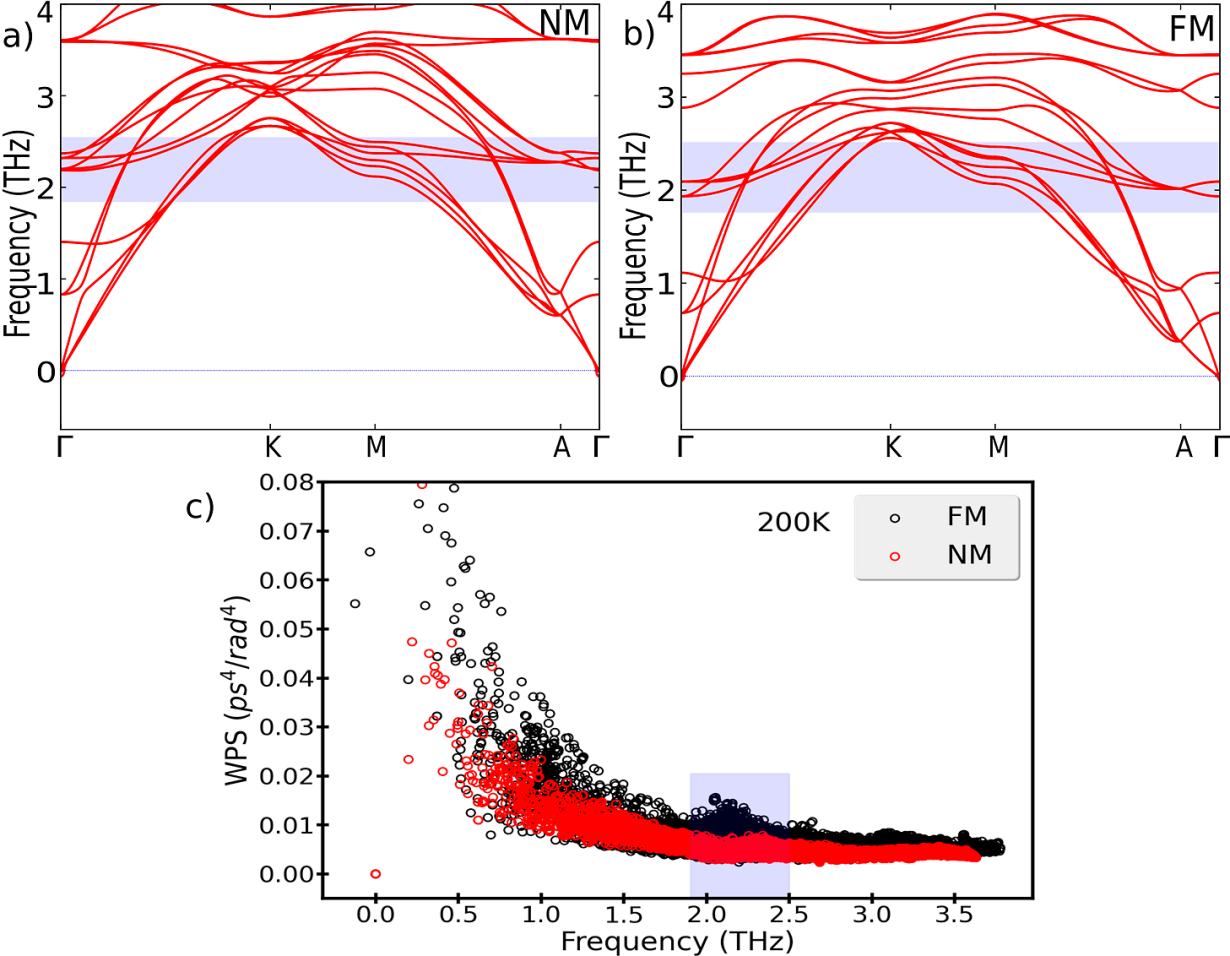
Phonon band diagrams for the magnetic (FM) (a) and non-magnetic (NM) (b) ordering and weighted phase space (WPS) available for three-phonon processes as a function of the frequency (c). The shaded area in (c) corresponds with the highlighted region in both band diagrams. The FM ordering shows an enhanced peak compared to the NM ordering in the WPS around 2.1 THz associated with Raman modes, leading to more scattering processes causing a reduction in the cross-plane thermal conductivity.

For understanding the reduction of κ_⊥_ in the magnetic phase, we have analyzed the phonon band structures and the weighted phase space (WPS) available for three-phonon processes for the NM and FM configurations (Figure 6). The WPS gives us an idea of the frequencies involved in phonon scattering processes that are different in the FM and NM states. From the phonon band structures, we can observe that the acoustic phonons undergo a shift toward lower frequencies, especially in the A–Γ path, related to a decrease in group velocities in the out-of-plane direction and hence in the thermal conductivity^33^ of the FM phase compared to the NM one discussed above.

Moreover, FM ordering shows an increase in the WPS, specifically a peak around 2 THz that is substantially different from the NM calculation. This peak is related to the phonon modes highlighted in Figure 6a,b and corresponds to two E_1*g*_ Raman active modes. In the magnetic phase, these modes are about 0.3 THz lower in energy, showing more crossings with the acoustic modes. In the NM calculation, two additional modes appear (an infrared-active A_2*u*_ mode and a higher-lying B_1*g*_ mode). These move up to about 3 THz in the FM calculation. The frequency lowering of the modes in the FM calculation leads to the observed additional scattering and causes a reduction in the thermal conductivity.

Strong coupling between Raman-active modes and a particular magnetic order has been reported in other two-dimensional magnets.^34^ Here, we observe that, together with the decrease in the group velocities of the acoustic modes, this coupling produces a considerable reduction of the lattice thermal conductivity in the magnetic phase of FGT.

## Conclusions

To summarize, we have combined experimental FDTR and ab initio calculations to demonstrate that the cross-plane thermal conductivity of 2D ferromagnet FGT presents a mixed contribution of diffusive and ballistic phonons. We have also shown that κ_⊥_ presents an abrupt reduction below the Curie temperature due to additional phonon scattering produced by a downshift in the frequency of acoustic and Raman-active optical phonons in the magnetic phase. Also, artificial stacking of a few layer thick FGT is a useful way of reducing the cross-plane thermal conductivity in this material.

## Experimental and Computational Details

Thermal conductivity was measured by a commercial FDTR from Fourier Inc. using a sinusoidally modulated pump laser (λ = 405 nm, *f* = 2 kHz to −50 MHz, 1 mW) and a continuous wave 532 nm probe laser (3 mW). Both lasers have Gaussian spot sizes 1/*e*^2^ with a radius of 3.7 or 10.5 μm. The probe beam is split before reaching the sample to work as a reference signal, improving the signal-to-noise ratio at low frequencies and compensating phase-shift offsets from beam paths and electronics. The same setup is described in detail in the ref (22). A 60 nm gold-thin film deposited by Ar plasma sputtering works as a reflective transducer. The fitting model considers Fourier heat conduction: the heat flux *q* = −κ∇*T*, where κ is a tensor to account for the material thermal conductivity anisotropy. The sample temperature is controlled inside a cold finger optical cryostat, down to 80 K. The whole stage is mounted on a piezoelectric table, which allows μm precision location of the laser spots on the sample. To promote the adhesion of FGT to the sapphire substrate, the samples were annealed under vacuum at 100 °C before the experiments.

κ_⊥_ in the magnetic and nonmagnetic phases of bulk FGT are calculated within a DFT^35,36^ framework using the VASP code.^37−39^ For all calculations, we performed a full relaxation of the structure (both atomic positions and lattice parameters were optimized) with a mesh of 16 × 16 × 3 *k*-points in the irreducible wedge of the Brillouin zone. The exchange–correlation potential chosen was the generalized gradient approximation in the Perdew–Burke–Ernzerhof scheme.^40^ The second-order interatomic force constants (IFCs) were determined using the Phonopy code^41,42^ in a 2 × 2 × 2 supercell with a *k*-mesh of 8 × 8 × 2 with no further relaxation of cell shape or volume. Third-order anharmonic IFCs were computed using the machinery of the ShengBTE code,^32^ considering interactions to third neighbors in a 2 × 2 × 2 supercell. The lattice thermal conductivity was calculated by solving the Boltzmann Transport Equation (BTE) for phonons by an iterative self-consistent method implemented in the ShengBTE code within a mesh of 36 × 36 × 8 *q*-points and a scale-broad parameter of 0.1.

## Supporting Information Available

Additional figures with X-ray powder diffraction pattern of exfoliated crystal,FTDR sensitivity analysis, and details of FDTR measurements with magnetic field (PDF)
