# Supplementary material for: Temperature and Thickness Dependence of the Thermal Conductivity in 2D Ferromagnet Fe3GeTe2
Source: ACS Appl Mater Interfaces. 2023 Oct 17;15(42):49538–44. doi: 10.1021/acsami.3c11578 (PMC10614195; doi:10.1021/acsami.3c11578)
Supplement: Supplementary file 1 — am3c11578_si_001.pdf [file am3c11578_si_001.pdf]

Supporting information of

Temperature and Thickness Dependence of the Thermal Conductivity in 2D Ferromagnet Fe_3GeTe_2

Marcel S. Claro,[*,†] Javier Corral-Sertal,[†] Adolfo Otero Fumega,[¶] Santiago Blanco-Canosa,[§,∥] Manuel Suárez-Rodríguez,[⊥] Luis E. Hueso,[⊥,∥] Victor Pardo,[*,#,@] and Francisco Rivadulla[†]

†CiQUS, Centro Singular de Investigacion en Quimica Bioloxica e Materiais Moleculares, Departamento de Quimica-Fisica, Universidade de Santiago de Compostela, Santiago de Compostela, E-15782, Spain.
¶Department of Applied Physics, Aalto University, Aalto, FI-00076 Finland
§Donostia International Physics Center (DIPC), San Sebastian, E-20018, Spain
∥IKERBASQUE, Basque Foundation for Science, Bilbao, E-48009, Spain
⊥CIC nanoGUNE BRTA, Donostia-San Sebastian, E-20018, Spain
#Departamento de Física Aplicada, Universidade de Santiago de Compostela, Santiago de Compostela, E-15782, Spain
@Instituto de Materiais iMATUS, Universidade de Santiago de Compostela, Santiago de Compostela, E-15782,
Spain

*E-mail: marcel.santos@usc.es; victor.pardo@usc.es

Nanometer-thick flakes (lateral size in the micron range) of Fe_3GeTe_2 studied in this work were mechanically exfoliated from millimeter-size crystals purchased from hq$^+$ graphene (https://www.hqgraphene.com/). The crystal structure and composition were confirmed by X-ray diffraction and EDS analysis; see Figure 1.

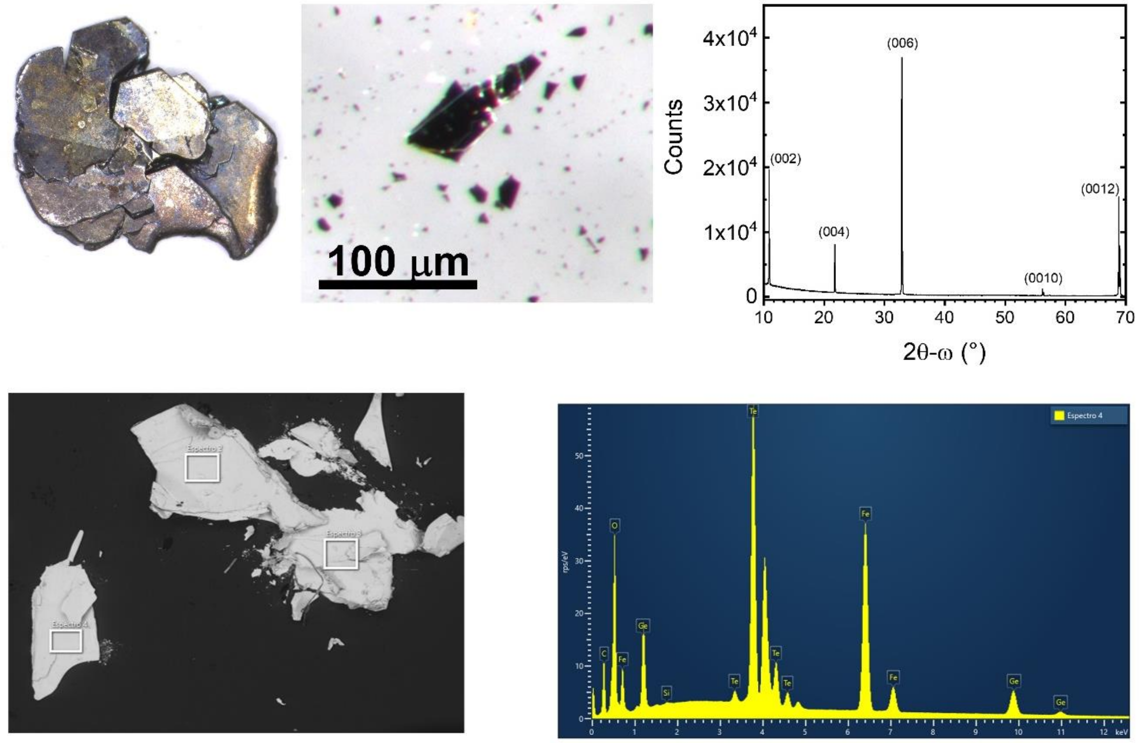

% mass	Fe	Ge	Te
Average experiment	32,7±1,0	14,7±0,3	51.52±1,7
Theoretical	33,82	14,66	51,52

Figure S1. Optical microscopy picture of one of the original pieces from which the flakes were exfoliated. The X-ray diffraction of the flakes shows only the (00l) peaks, confirming the orientation //ab of the flakes. Bottom: scanning electron microscopy image of some flakes exfoliated from a larger piece and Energy Dispersive X-ray spectroscopy (EDS) analysis, confirming the Fe_3GeTe_2 stoichiometry.

For the mechanical exfoliation of flakes with thicknesses between 10- 250 nm, we used low residue adhesive tape BT-150E-CM from Nitto. The flakes were then transferred to a PDMS stamp, and from there to a cleaned substrate of sapphire.

Dozens of flakes were inspected and first selected by optical microscopy; the thickness of the selected flakes was measured by Atomic Force Microscopy (AFM); Figure S2 and S3.

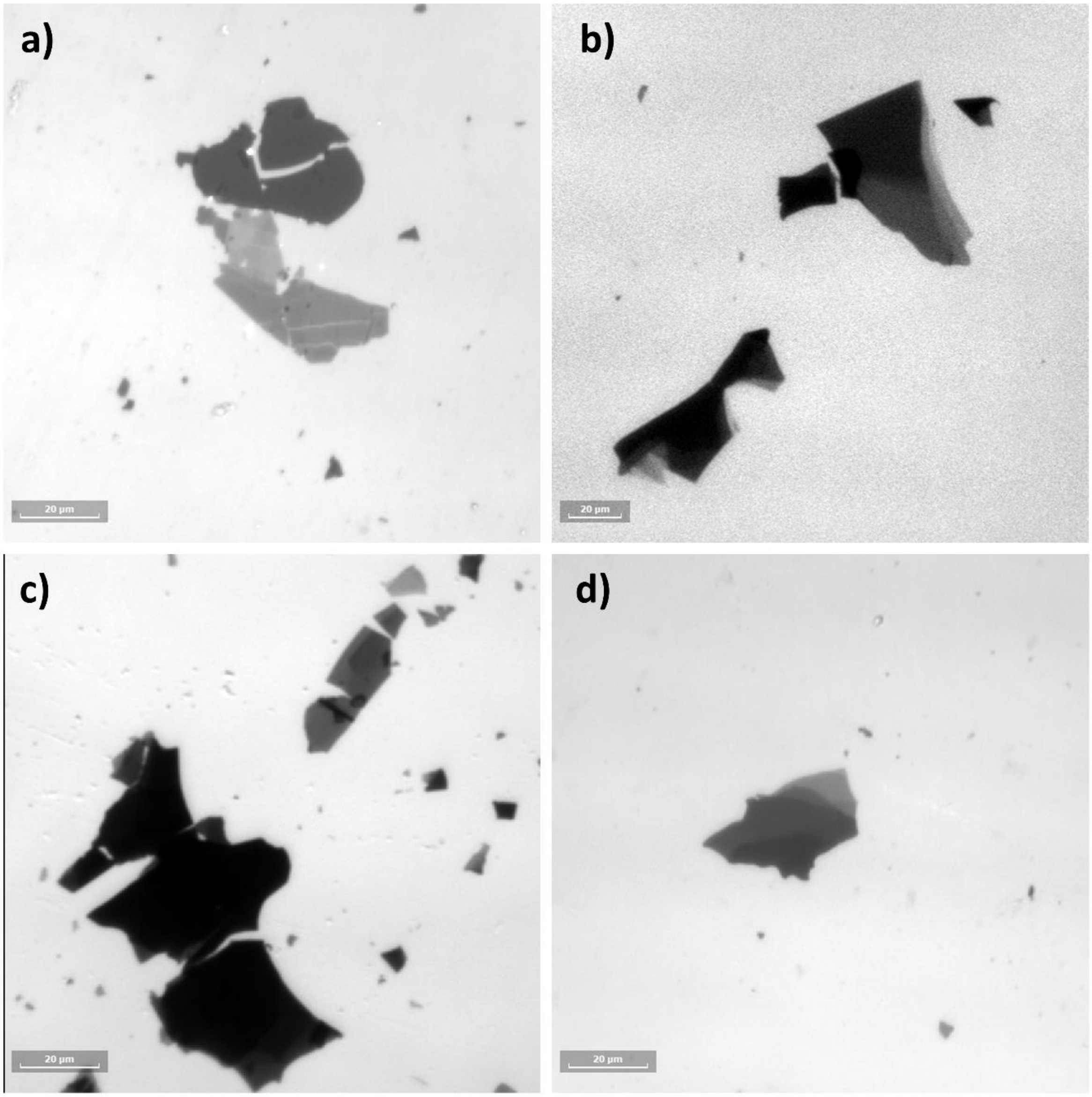

Figure S2. Optical microscopy pictures of flakes with different thickness studied in this work. The scale bar is 20 microns. A preselection of the flakes to be measured by AFM is done by measuring the contrast in the optical image.

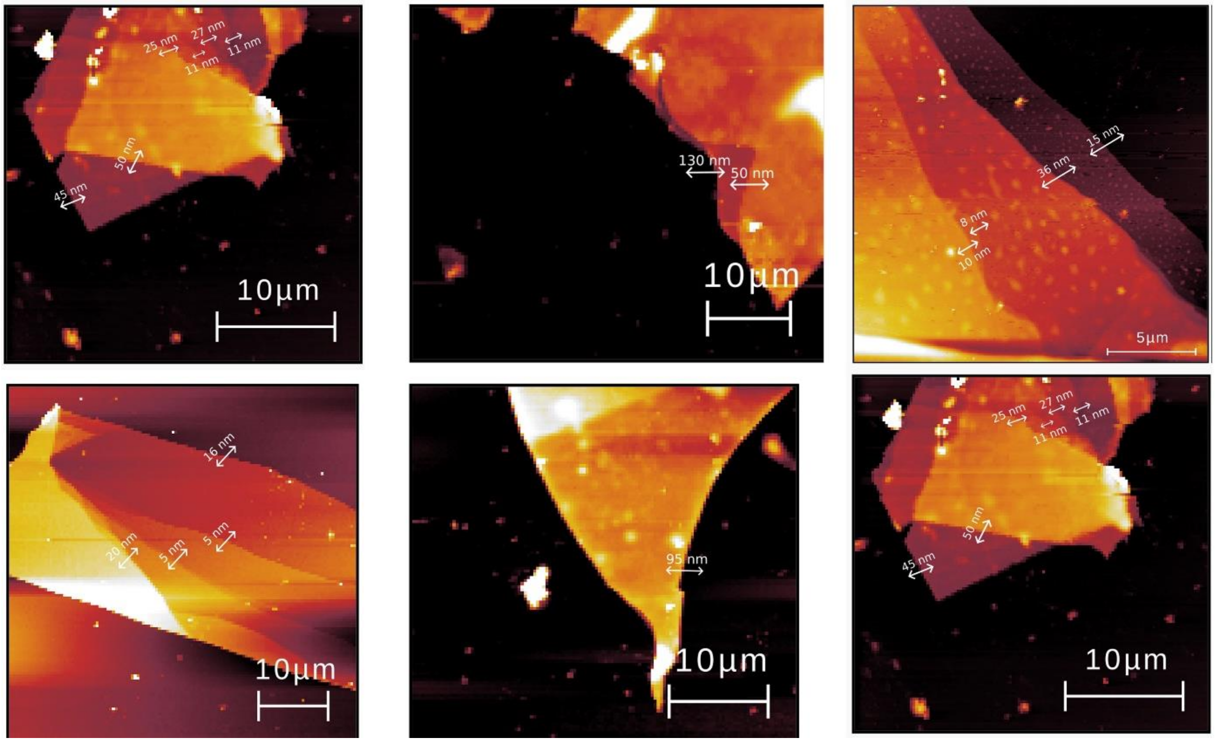

Figure S3. AFM images of some of the flakes with different thickness, transferred to the sapphire substrate, studied in this work. Some of the flakes show several layers, which allow the study of the thermal conductivity as a function of thickness thickness without varying the sample/substrate interface.

For the thermal conductivity experiments, we selected flakes free of defects that show a homogeneity over several square microns. We picked several samples, of different batches, covering a range of thicknesses from ≈20 to ≈250 nm (Figures S2 and S3).

The cross-plane thermal conductivity was measured by Frequency Domain Thermoreflectance (FDTR).[1] FDTR is a non-contact optical pump–probe techniques, in which one beam of light (the pump) acts as a heat source while a second beam (the probe) detects the resulting temperature change (ΔT) through a change in surface reflectivity (ΔR).

$$\Delta T = \left(\frac{dR}{dT}\right)^{-1} \Delta R = (\beta)^{-1}\Delta R$$

The variable heat source produce temperature gradients and transients which makes it able to measure κ, c and thermal boundary conductance with good precision.

In our setup, a sinusoidal modulated pump laser (λ=405 nm, modulating f=2kHz–50 MHz, Gaussian spot sizes $1/e^2$ radius ≈ 3.7 or 10.5 μm) is focused on the surface of the film, coated by a 60-nm-thick layer of Au, to produce an oscillatory modulation of the surface temperature. This results in a periodic variation of the Au thermoreflectance, which is probed by a laser beam (λ=532 nm); see Figure S4. The probe beam is split before reaching the sample to work as a reference signal, improving the signal-to-noise ratio at low frequencies and compensating phase-shift offsets from beam paths and electronics. The same setup is described in detail in reference [2].

The thermal properties of the sample are obtained by fitting the phase data to an analytical solution of the heat diffusion equation, in a multilayer model as described below. The thermal conductivity of the substrates was obtained from ref.[3]; for Au we measured the electrical conductivity in co-deposited samples, and used the Wiedemann-Frantz law to obtain its thermal conductivity. The C_p of the substrate and Au transducer were obtained from the literature.

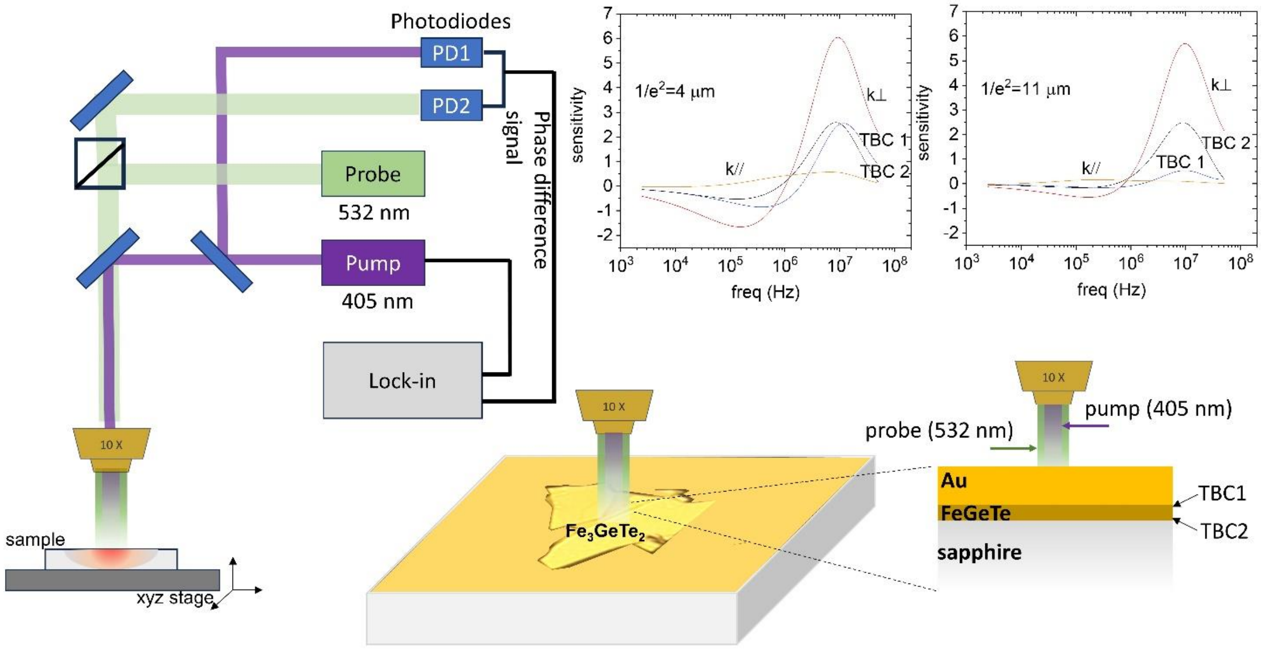

Figure S4. Simplified scheme of the FDTR setup and detail of the measurement of the flakes, with the different thermal boundary conductance (TBCs) used in the model. The two graphs represent the sensitivity of the measurement to TBC1, TBC2 and the in-plane ($k_{//}$) and cross-plane ($k_{\perp}$) thermal conductivity of Fe_3GeTe_2, according to equation (1). The parameters used for the sensitivity analysis are TBC1=40 MW $m^{-2}K^{-1}$; TBC2=25 MW $m^{-2}K^{-1}$; and $\kappa_{\perp}$=0.75 W $m^{-1}K^{-1}$. For $k_{//}$ we varied the values from 1-100 W $m^{-1}K^{-1}$. The line presented in the plots corresponds to 100 W $m^{-1}K^{-1}$, where the largest sensitivity to this parameter (although still very small) was achieved. The other parameters, like the C_p of Au, sapphire and Fe_3GeTe_2, are taken from the literature. The thermal conductivity of Au and sapphire were measured in an independent experiment. The thickness of the Au transducer layer was determined by x-ray reflectivity and was kept fixed in all the experiments.

FTDR fitting model:

There are several analytical models which can provide reliable amplitude and phase shift required for the parameters fitting. Most of them consider Fourier heat conduction, i.e., the heat flux q is given by.

$$q = -\kappa \nabla T \quad (1)$$

where κ is a constant, or a tensor to account for the material anisotropy.

$$\kappa = \begin{pmatrix} \kappa_{xx} & \kappa_{xy} & \kappa_{xz} \\ \kappa_{yx} & \kappa_{yy} & \kappa_{yz} \\ \kappa_{zx} & \kappa_{zy} & \kappa_{zz} \end{pmatrix} \qquad (2)$$

Due to symmetry, this tensor can be reduced to a diagonal matrix in cubic materials:

$$\kappa = \begin{pmatrix} \kappa_{\parallel} & 0 & 0 \\ 0 & \kappa_{\parallel} & 0 \\ 0 & 0 & \kappa_{zz} \end{pmatrix} \quad (3)$$

Or to a symmetric matrix in hexagonal unit cells (Wurtzite and several vdW materials):

$$\kappa = \begin{pmatrix} \kappa_{xx} & \kappa_{xy} & 0 \\ \kappa_{yx} & \kappa_{yy} & 0 \\ 0 & 0 & \kappa_{zz} \end{pmatrix} \quad (4)$$

The energy conservation in the Fourier scheme is given by.

$$c\frac{\partial T}{\partial t} = -\nabla \cdot q + f \quad (5)$$

Where *f* is a heat internal source.

When the system is formed of continuous thin films, several simplifications can be done. Then, for each layer the temperatures at top and bottom interfaces are given by:

$$\begin{pmatrix} T_{bottom} \\ q_{bottom} \end{pmatrix} = M(\omega, c, \kappa, t) \begin{pmatrix} T_{top} \\ q_{top} \end{pmatrix} \quad (6)$$

To impose total energy conservation and energy transfer between layers a transfer matrix is usually used.

$$\begin{pmatrix} T_{bottom,last} \\ q_{bottom,last} \end{pmatrix} = M_1(\omega, c_1,\ \kappa_1, t_1) \cdot M_2(\omega, c_2,\ \kappa_2, t_2) \cdots M_{last}(\omega, c_{last},\ \kappa_{last}, t_{last}) \begin{pmatrix} T_{top,1} \\ q_{top,1} \end{pmatrix} (7)$$

The matrix $M_n(\omega, c_n,\ \kappa_n, t_n)$depends on ω, the layer thickness (t_n), and the parameter c_n and $\kappa_n$of the material forming this layer as shown in the Figure S5.

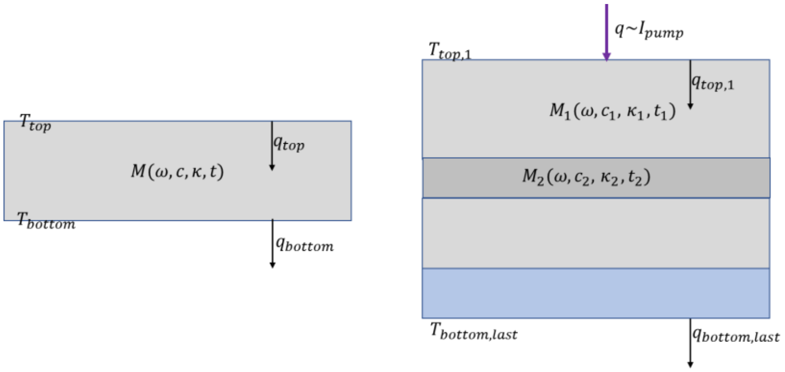

Figure S5. The Transfer matrix formulation of multi-layered thin films.

To account for the interface thermal resistance (inverse of TBC), also called Kapitza Resistance,

$$h_{n,n+1}(T_n - T_{n+1}) = q_{\perp} \quad (8)$$

Another matrix is added for each interface:

$$M_{n,n+1} = \begin{pmatrix} 1 & -1/h_{n,n+1} \\ 0 & 1 \end{pmatrix} \quad (9)$$

Since we are interested in the function of $T_{top,1}$, this system can be solved considering that $q_{top,1}$ is the power absorbed from the pump laser ($q_{top,1} = I_{pump}$), and that $q_{bottom,last} = 0$, $T_{bottom,last} = constant$, which means that the substrate is thick enough to diffuse the heat before it reaches the opposite side.

If

$$\begin{pmatrix} \Theta_{00} & \Theta_{01} \\ \Theta_{10} & \Theta_{11} \end{pmatrix} = M_1(\omega, c_1, \kappa_1, t_1) \cdot M_1(\omega, c_2, \kappa_2, t_2) \cdots M_{last}(\omega, c_{last}, \kappa_{last}, t_{last}) \quad (10)$$

Then,

$$\begin{pmatrix} T_{bottom,last} \\ 0 \end{pmatrix} = \begin{pmatrix} \Theta_{00} & \Theta_{01} \\ \Theta_{10} & \Theta_{11} \end{pmatrix} \begin{pmatrix} T_{top,1} \\ q_{top,1} \end{pmatrix} \quad (11a)$$

$$\Theta_{10} T_{top,1} = -\Theta_{11} q_{top,1} \quad (11b)$$

Which relates the heat pumped in the surface and the temperature measured by the FTDR system.

Gaussian laser beams focused on the surface are also usually assumed, as well as concentric pump and probe beams. The beam intensities are then:

$$I_{pump}(r,t) = A_{pump} \frac{2}{\pi r_{pump}^2} \exp\left(\frac{-2r^2}{r_{pump}^2}\right) e^{-i\omega_0 t} \quad (12a)$$

$$I_{probe}(r) = A_{probe} \frac{2}{\pi r_{probe}^2} \exp\left(\frac{-2r^2}{r_{probe}^2}\right) \quad (12b)$$

$T_{top,1}$ can be represented as a phasor (complex-number): $A(\omega)e^{-i\omega t+\varphi(\omega)}$, which is easily correlated with the Fourier Transform $\Im = \mathcal{F}\{T_{top,1}\}$

$$A(\omega) = \|\Im\| \text{ and } \varphi(\omega) = \arctan\left(\frac{Imag(\Im)}{Re(\Im)}\right) \quad (13)$$

With all these considerations, using cylindrical symmetry and Hankel transforms, the Fourier Transform of the temperature is.

$$\Im(r,\omega) = A_{pump}\delta(\omega - \omega_0) \int_0^\infty \chi \left(\frac{-\Theta_{11}}{\Theta_{10}}\right) \exp\left(\frac{-\chi^2({r_{pump}}^2 + {r_{probe}}^2)}{8}\right) d\chi \quad (14)$$

And each matrix is:

$$M_n = \begin{pmatrix} \cosh(\mu_n t_n) & {\kappa_{zz}}^{-1}\sinh(\mu_n t_n) \\ -\kappa_{n,zz}\sinh(\mu_n t_n) & \cosh(\mu_n t_n) \end{pmatrix}, \; {\mu_n}^2 = \frac{\kappa_{n,\parallel}\chi^2 + i c_n \omega}{\kappa_{n,zz}} \quad (15)$$

Since the actual measurement in an FDTR experiment is the reflectivity of the probe beam ($\beta\Delta T$), the probe beam averages the result over the spot area. If the detector responsivity is R (in V/W), the Fourier transform of the signal measured in the detector, S, is

$$\mathcal{F}\{S(t)\} = R\beta \int_0^\infty I_{probe}(r) \Im(r,\omega) dr \quad (16)$$

S is a phasor, and the amplitude and phase are given in the FDTR lock-in output.

We performed sensitivity analysis for estimating the uncertainty of our FDTR measurements. We define the sensitivity of the phase signal to a fitting parameter α as (the phase in radians):[1]

$$S_{\alpha} = \frac{d\ \varphi}{d\ ln\alpha} \qquad (17)$$

To decouple the sensitivity for the parameters of interest ($k_{\perp}$, G1 and G2) we combined experiments with different spot sizes, between 1 MHz to 50 MHz (see Figure S3).

However, due to the strong correlation between the G1 and G2 parameters, in practice, only the sum G1+G2 is considered in the fitting. Figure S5 shows the variation of this parameter with temperature.

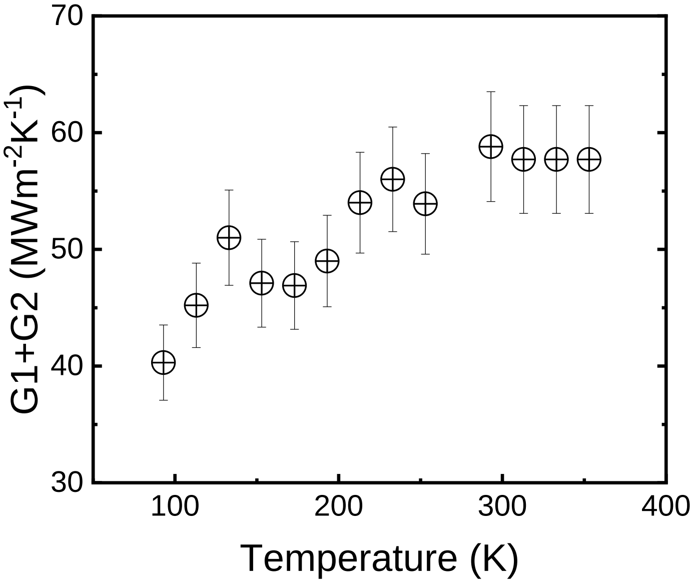

Figure S6. Total thermal boundary conductivity (TBC) G1+G2 TBCs extracted from the FTDR phase-shift fittings.

For measuring the thermal conductivity of the flakes under an external magnetic field, we have used permanent magnets made of NdFeB (N45), of toroidal shape. These magnets can be placed and removed keeping the measured spot fixed. The toroidal shape allows the transmission of the pump and probe laser without any interference; see Figure S6.

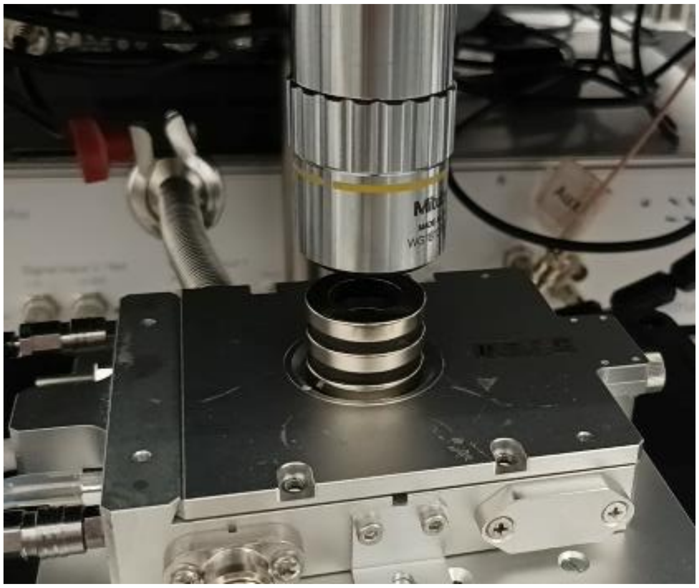

Figure S7. Photograph of the sample inside the optical cryostat below the FDTR confocal lenses and added toroidal magnets, for measurements with a magnetic field.

The toroidal magnets create a considerably uniform and constant out-of-plane magnetic field. Since magnetization and geometry are well known, the field was determined using Finite Element Method (Software: FEMM 4.2) (Figure S7) from which we estimate a field of 56 mT in the sample plane.

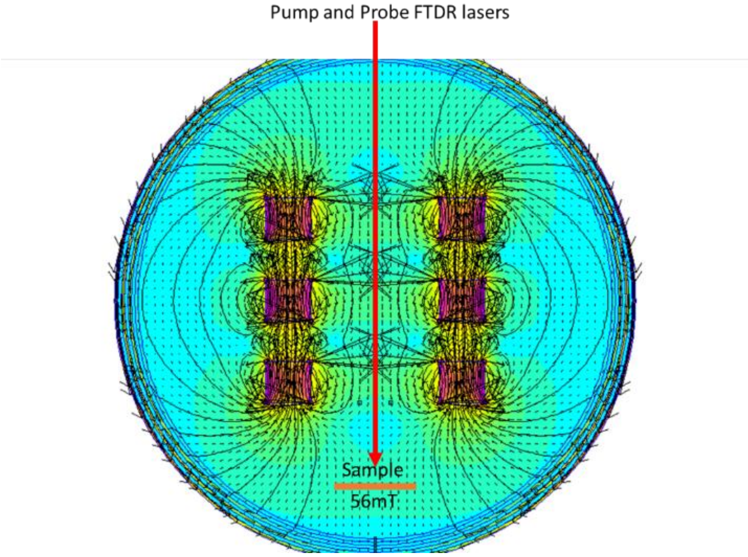

Figure S8. Finite-Element simulation of the Magnetic Field applied to the sample during the FDTR experiment with toroidal permanent magnets.

Stacked flakes:

The temperature dependence of $k_{\perp}$ in the region of superposition of the two flakes (point 3 in Figure 3 of the main text) is shown in Fig. S9. In this case, $\kappa_{\perp}$ remains very small, with a weak temperature dependence in the whole interval, without any substantial change around T_C, suggesting that in this case the flakes act as independent resistances with a strong dependence on thermal resistance (TBC) between the two flakes of FGT.

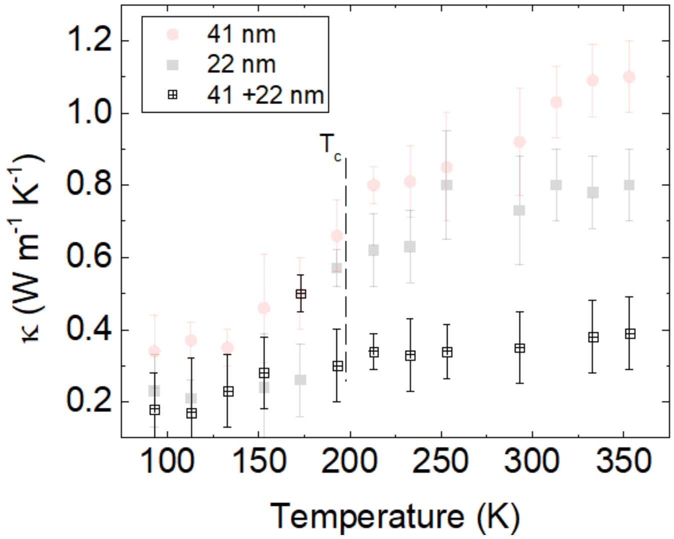

Figure S9: Experimental temperature dependence of the thermal conductivity of the two flakes (22 nm and 41 nm) superposition corresponding to point 3 in Fig. 3a).

A similar trend was observed on other stacked flakes e.g. the stacked layer presented below in Fig. S10 has a flake of 14 nm stacking over a 20 nm flake. Once more, the best fitting is obtained considering two FGT flakes and TBC of 22 MWm^2K^{-1}. This TBC is like the TBC of the 41+22 nm sample, considering sample preparation and fitting variability. Therefore, we have neither observed any evident dependence of $k_{\perp}$, or TBC, on the relative orientation of the superposed FGT flakes on other few samples. Although further experiments with more samples should be performed to fully confirm this result.

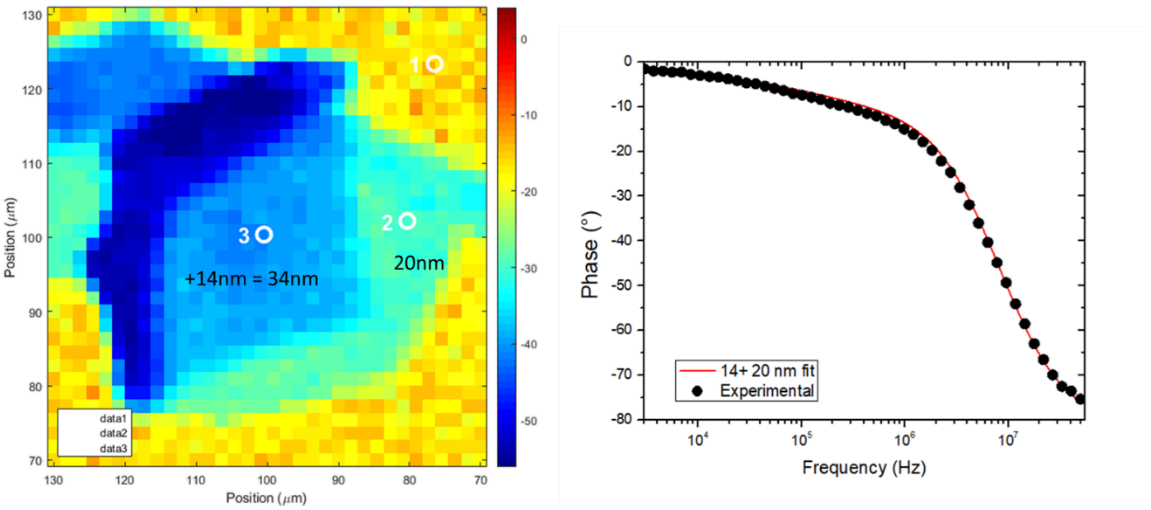

Figure S10. Phase-shift map at 20 MHz of the stacked flakes (left). In this image the flakes are already covered with 40 nm of Au for FDTR measurements. Marked points 1 (substrate) and 2 (20 nm thick flake) are used as reference to fix related parameters in model. And the measured phase-shift of stacked flakes at point 3 (right). In red, a fitting using 2 flakes separated by a TBC of 22 MWm^2K^{-1} is represented.

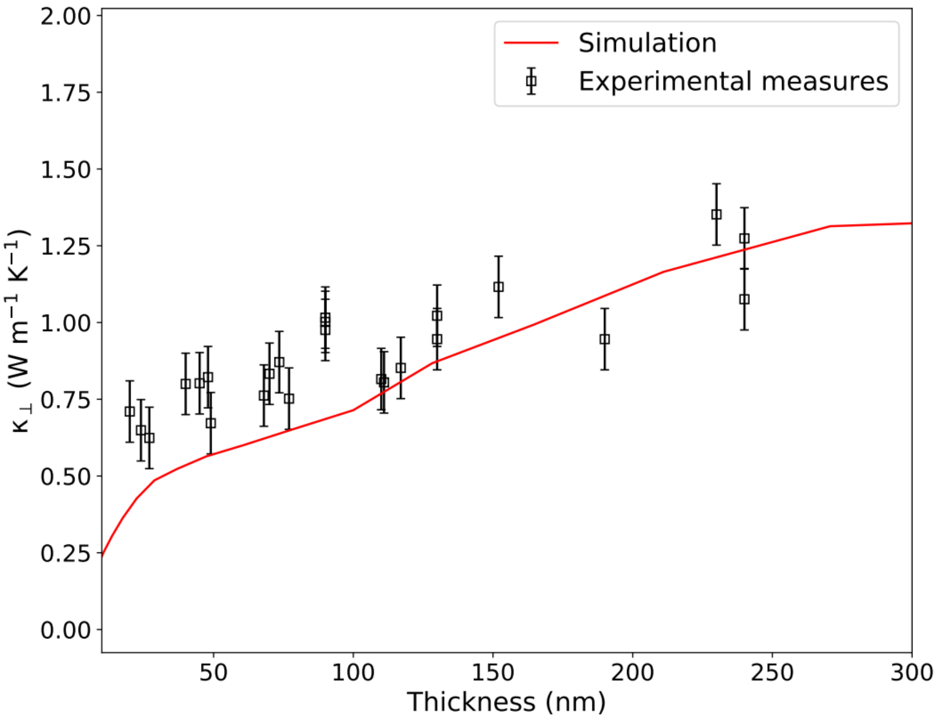

Figure S11. Comparison between experimental $\kappa_\perp$ (Figure 4a) and accumulated $\kappa_\perp$ of FM phase from DFT calculations (Zoomed from Figure 4c).

References

(1) Schmidt, A. J.; Cheaito, R.; Chiesa, M. A Frequency-Domain Thermoreflectance Method for the Characterization of Thermal Properties. *Rev. Sci. Instrum.* **2009**, *80* (9). https://doi.org/10.1063/1.3212673.

(2) Yang, J.; Maragliano, C.; Schmidt, A. J. Thermal Property Microscopy with Frequency Domain Thermoreflectance. *Rev. Sci. Instrum.* **2013**, *84* (10). https://doi.org/10.1063/1.4824143.

(3) Langenberg, E.; Ferreiro-Vila, E.; Leborán, V.; Fumega, A. O.; Pardo, V.; Rivadulla, F. Analysis of the Temperature Dependence of the Thermal Conductivity of Insulating Single Crystal Oxides. *APL Mater.* **2016**, *4* (10). https://doi.org/10.1063/1.4966220.

(4) Kohn, W.; Sham, L. J. Self-Consistent Equations Including Exchange and Correlation Effects. *Phys. Rev.* **1965**, *140* (4A), A1133. https://doi.org/10.1103/PhysRev.140.A1133.

(5) Hohenberg, P.; Kohn, W. Inhomogeneous Electron Gas. *Phys. Rev.* **1964**, *136* (3B), B864. https://doi.org/10.1103/PhysRev.136.B864.

(6) Kresse, G.; Furthmüller, J. Efficient Iterative Schemes for *Ab Initio* Total-Energy Calculations Using a Plane-Wave Basis Set. *Phys. Rev. B* **1996**, *54* (16), 11169. https://doi.org/10.1103/PhysRevB.54.11169.

(7) Kresse, G.; Furthmüller, J. Efficiency of Ab-Initio Total Energy Calculations for Metals and Semiconductors Using a Plane-Wave Basis Set. *Comput. Mater. Sci.* **1996**, *6* (1), 15–50. https://doi.org/10.1016/0927-0256(96)00008-0.

(8) Kresse, G.; Hafner, J. *Ab Initio* Molecular Dynamics for Liquid Metals. *Phys. Rev. B* **1993**, *47* (1), 558–561. https://doi.org/10.1103/PhysRevB.47.558.

(9) Perdew, J. P.; Burke, K.; Ernzerhof, M. Generalized Gradient Approximation Made Simple. *Phys. Rev. Lett.* **1996**, *77* (18), 3865. https://doi.org/10.1103/PhysRevLett.77.3865.

(10) Togo, A. First-Principles Phonon Calculations with Phonopy and Phono3py. *J. Phys. Soc. Japan* **2023**, *92* (1). https://doi.org/10.7566/JPSJ.92.012001.

(11) Togo, A.; Tanaka, I. First Principles Phonon Calculations in Materials Science. *Scr. Mater.* **2015**, *108*, 1–5. https://doi.org/10.1016/j.scriptamat.2015.07.021.

(12) Li, W.; Carrete, J.; Katcho, N. A.; Mingo, N. ShengBTE: A Solver of the Boltzmann Transport Equation for Phonons. *Comput. Phys. Commun.* **2014**, *185* (6), 1747–1758. https://doi.org/10.1016/J.CPC.2014.02.015.
